# Carelizumab and rivoceranib for advanced extramammary Paget’s disease: an investigator-initiated multicenter, single-arm, phase II trial

**DOI:** 10.1093/oncolo/oyaf202

**Published:** 2025-07-31

**Authors:** Sheng Zhang, Mengyang Ju, Qin Wang, Dongyuan Zhu, Zengjun Liu, Xiongjun Ye, Wenfeng Li

**Affiliations:** Medical Oncology, Shanghai Cancer Center and Department of Oncology, Shanghai Medical College, Fudan University, Shanghai 200030, China; Medical Oncology, Shanghai Cancer Center and Department of Oncology, Shanghai Medical College, Fudan University, Shanghai 200030, China; Shanghai University of Engineering Science, Shanghai 201620, China; Rare Tumors Department, Shandong Cancer Hospital and Institute, Shandong First Medical University and Shandong Academy of Medical Sciences, Jinan 250117, China; Rare Tumors Department, Shandong Cancer Hospital and Institute, Shandong First Medical University and Shandong Academy of Medical Sciences, Jinan 250117, China; Department of Urology, National Cancer Center/National Clinical Research Center for Cancer/Cancer Hospital, Chinese Academy of Medical Sciences and Peking Union Medical College, Beijing 100021, China; Affiliated Hospital of Qingdao University, Qingdao 266003, China

**Keywords:** carelizumab, rivoceranib, clinical trial, extramammary Paget’s disease

## Abstract

**Background:**

Extramammary Paget’s disease (EMPD) is a rare cancer without standard therapy. We evaluated the safety and efficacy of a combination of the humanized monoclonal antiprogrammed death-1 (PD-1) antibody, carelizumab, with rivoceranib, a vascular endothelial growth factor receptor (VEGFR) inhibitor, in patients with advanced EMPD.

**Methods:**

This multicenter, single-arm, phase II trial included 12 patients with EMPD. The primary endpoint is the objective response rate (ORR), defined as the proportion of patients achieving complete response (CR) or partial response (PR) according to RECIST v1.1 criteria. It is hypothesized that the ORR will be less than 10% if the treatment is ineffective and will reach 35% if effective. Secondary endpoints include progression-free survival (PFS) and safety assessments. Eligible patients are aged ≥18 years, with histologically confirmed advanced EMPD. Treatment consists of carelizumab (200 mg intravenously every 3 weeks) and rivoceranib (250 mg orally once daily), with dose adjustments permitted based on toxicity profiles.

**Results:**

Between June 2024 and February 2025, 12 patients were enrolled. One patient withdrew from the trial because of nontreatment-related accidents, and a total of 11 patients completed at least 1 cycle of treatment. The median PFS is 2.4 months (95% CI, 2.1-4.4 months). Among these 11 patients, no patients achieved CR or PR. Six patients (50%) experienced grade 3 adverse events.

**Conclusions:**

This is the first trial evaluating the combination of a PD-1 inhibitor and a VEGFR inhibitor in advanced EMPD. This combination therapy is well tolerated but does not appear to have clinically meaningful activity in advanced EMPD. This trial was registered at the Chinese Clinical Trial number registration: 2400086153.

**Key Points**

- Treatment options for patients with advanced Extramammary Paget’s disease, a rare skin cancer, are scarce.
- This phase II trial evaluated the efficacy of the combination of PD1 inhibitors and VEGFR inhibitors for the first time.
- No efficacy was found in this trial, suggesting further exploration should focus on biomarker selection in the future.

## Trial information

**Table:** 

**Disease**	Advanced Extramammary Paget’s disease
**Stage of Disease**	IV
**Prior Therapy**	No limitation.
**Type of Study**	Phase II
**Primary Endpoints**	objective response rate (ORR), defined as the proportion of patients achieving complete response (CR) or partial response (PR)according to RECIST v1.1 criteria.
**Secondary Endpoints**	progression-free survival (PFS) and safety assessments.
**Additional Details of Endpoints or Study Design** The study was reviewed and approved by the Institutional Review Board of the Shanghai Cancer Center, Fudan University, and all participating centers. The trial was conducted in accordance with the International Conference on Harmonization Good Clinical Practice guidelines and the Declaration of Helsinki. All participating patients provided written informed consent. Twelve patients were enrolled. One patient withdrew from the trial because of nontreatment-related accidents, and a total of 11 patients completed at least 1 cycle of treatment.	

## Drug information

**Table:** 

**Generic/Working name**	Carelizumab and Rivoceranib
**Company Name**	Hengrui Pharmaceuticals, China
**Drug Type**	Carelizumab is an anti-PD-1 monotherapy. Rivoceranib is a VEGFR inhibitor.
**Drug Class**	Carelizumab is an ICI. Rivoceranib is a targeted therapy
**Dose**	Carelizumab 200 mg + Rivoceranib 250 mg
**Route**	Carelizumab is intravenous (IV). Rivoceranib is oral.

## Patient characteristics

**Table:** 

**Median age (range)**	69.5 (60-86)
**Number of Patients, Female/Female**	11/1
**Stage**	IV (100%)
**Location of primary tumor**	
Scrotum	8(67%)
Vulva	3(8%)
Penis	1(25%)
**Prior Surgery**	
Yes	9(75%)
No	3(25%)
**Prior Radiotherapy**	
Yes	6(50%)
No	6(50%)
**Number of Prior Chemotherapy**	
0-2	9(75%)
>2	3(25%)
**Metastatic site(s)**	
Inguinal lymph nodes	12(100%)
Nonlnguinal lymph nodes	8(67%)
Lung	4(34%)
Liver	6(50%)
Bone	7(58)
Yes	9(75%)
No	3(25%)
**Prior Radiotherapy**	
Yes	6(50%)
No	6(50%)
**ECOG Score**	
0	3(25%)
1	9(75%)
**PD-L1 Positive**	1(9%)
**TMB High**	0

## Primary assessment method

**Table:** 

**Statistical Analysis**	It is hypothesized that the ORR will be less than 10% if the treatment is ineffective and will reach 35% if effective. Simon’s MinMax 2-stage design was employed, with a power of 80% and a significance level (α) of 0.05. In the first stage, 11 patients were enrolled. If 2 or more patients achieve ORR, the study will proceed to the second stage, where an additional 7 patients will be enrolled, bringing the total to 18 patients. If a combined total of 5 patients achieves ORR across both stages, the study will be considered successful.
**Number of Patients Screened**	18
**Number of Patients Enrolled**	12
**Number of Patients Evaluable for Toxicity**	11
**Number of Patients Evaluated for Efficacy**	11
**Evaluation Method**	The response of patients with measurable target lesions on CT imaging was evaluated according to RECIST version 1.1. The efficacy of those with skin lesions but deemed as not measurable was evaluated according to WHO criteria. Disease progression was defined as the deterioration of the subject’s condition caused by the indication of the study. The progression of imaging and clinical symptoms and signs was included. Disease progression was considered to be the presence of a new metastasis relative to the primary tumor or the progression of an existing metastasis.
**Response assessment, CR/PR**	0/0
**Response assessment, SD/PD**	0/11
**Outcome Notes** Response assessment: Figure 1.	

**Figure 1.**
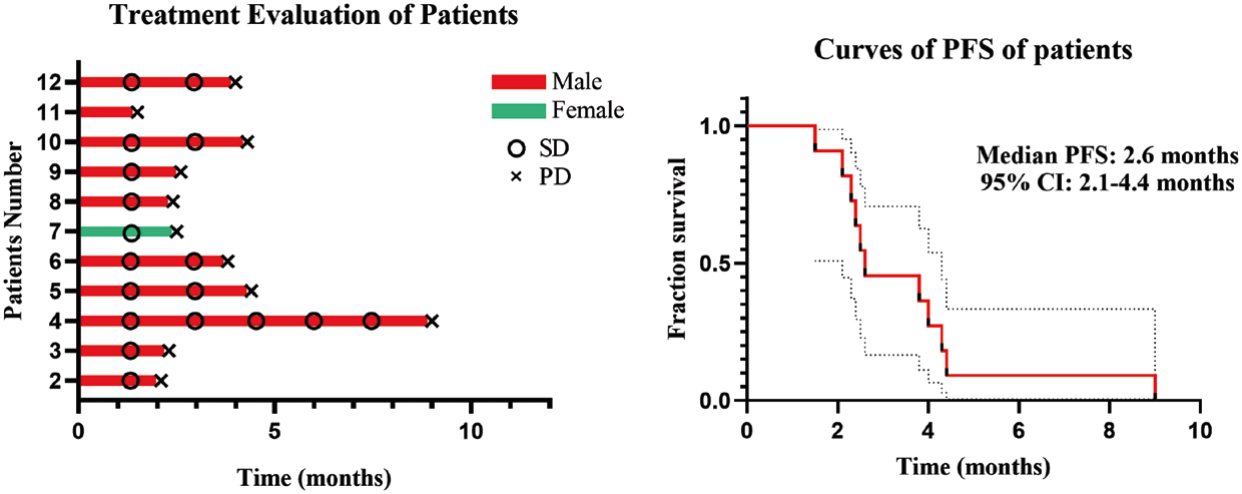
(A) The timeline of the treatment evaluation among 11 patients who completed at least 2 cycles. (B) PFS of the 11 patients who completed at least 2 cycles. Abbreviations: PD, progression disease; SD, stable disease.

## General toxicity profile

Common and grade 3 or higher adverse events observed with the combination therapy

**Table:** 

Adverse event	Grade 1	Grade 2	Grade 3	%
Fatigue	3	5	1	82
Nausea	1	1	0	18
Vomiting	2	1	0	27
Abdominal pain	1	1	0	18
Diarrhea	1	2	0	27
Skin and subcutaneous tissue disorders	2	4	1	64
Constipation	0	2	0	18
Cough	0	1	0	9
Dry skin	2	1	1	36
Dyspnea	0	0	0	0
Edema limbs	0	0	0	0
Flank pain	1	1	0	18
Flu-like symptoms	0	0	0	0
Musculoskeletal and connective tissue disorder	1	2	0	27
Noncardiac chest pain	0	1	0	9
Anemia	0	0	0	0
Hypertension	4	2	2	73
Sciatic pain	0	0	0	0
Tachycardia	3	1	1	45

## Discussion

Extramammary Paget’s disease (EMPD) is a rare epithelial malignancy, primarily occurring in the skin with rich apocrine glands, including vulva, scrotum, penis, and axilla.^1^ EMPD tends to be indolent and is often identified as carcinoma in situ at the time of diagnosis.^2^ However, when distant metastases occur, 5-year survival rates are <10%.^3^ For now, there have not been established treatments for metastatic EMPD, and treatment experience is based on case reports or small case series. Chemotherapy regimens, such as docetaxel, fluoropyrimidines, S-1, platins, and their combination therapy, are attempted and achieve limited progress.^4^ In general, the treatment efficacy is limited with the risk of adverse effects. Therefore, it is essential to find more effective and tolerable treatment options for advanced EMPD.

The programmed death-1 (PD-1)/PD-L1 pathway is crucial for the normal physiological regulation and suppression of immune responses in healthy tissues. Numerous tumors manage to evade immune detection and attack by increasing their PD-L1 expression. Humanized monoclonal antibodies targeting PD-1 and PD-L1 have demonstrated both safety and efficacy in treating a variety of cancers.^5^ We noticed several researchers have published case reports about the role of immune-checkpoint-inhibitors monotherapy in patients with EMPD.^6-8^ But the conclusions are conflicting and there is still a lack of clinical trials to provide higher-quality evidence. Furthermore, the treatment landscape for the combination of ICI and vascular endothelial growth factor receptor (VEGFR) inhibitor for EMPD remains underexplored, particularly there has been substantial evidence that the combination has a significant synergistic anticancer effect in various kinds of solid tumors.^9^ Research indicates that combining carelizumab, with rivoceranib, significantly decreases peripheral blood regulatory T cell (Treg) levels and increases the effector T cell (Teff) to Treg ratio. Additionally, the combination appears to enhance objective tumor response rates while maintaining the durability of immunotherapy. Consequently, numerous clinical trials have been conducted on this combination therapy, targeting advanced cancers such as hepatocellular carcinoma, lung cancer, intrahepatic cholangiocarcinoma, gastric adenocarcinoma, triple-negative breast cancer, and other solid tumors. These studies have investigated the safety, tolerability, and preliminary efficacy of camrelizumab in combination with rivoceranib across various malignancies.^10-12^

**Figure 2.**
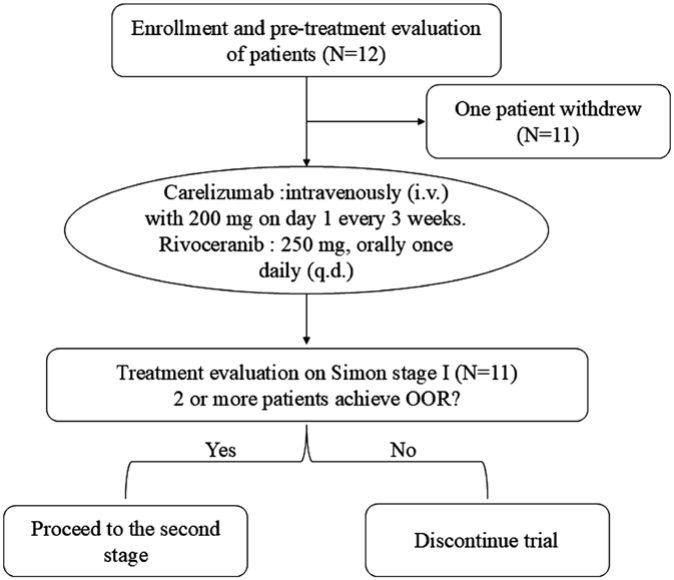
Study schema.

Thus, we hypothesized the combination of the PD-1 antibody, carelizumab, with the VEGFR inhibitor, rivoceranib, would be a potential strategy for patients with advanced EMPD. To our knowledge, this is the first clinical trial to investigate the objective response rate (ORR) and safety profile of this combination therapy in patients with advanced EMPD. Figure 2 shows the study schema.

The primary endpoint is the ORR, defined as the proportion of patients achieving complete response or partial response according to RECIST v1.1 criteria. Secondary endpoints include progression-free survival (PFS) and safety assessments. Patients receive carelizumab intravenously (i.v.) with 200 mg on day 1 every 3 weeks. The administration regimen for rivoceranib is as follows: 250 mg, orally once daily (q.d.), to be taken within half an hour after a meal, with continuous dosing. The treatment cycle is for 3 weeks. If tolerated, the dose can be escalated to a maximum of 500 mg q.d. Treatment was continued until documented disease progression, development of unacceptable level of toxic effects, withdrawal of consent, or decision by investigator to discontinue treatment.

From June 2024 to March 2025, 12 patients were enrolled (see Patient characteristics table). Median age was 69.5 years (range 60-86 years). 11 patients (92%) are male, and 1 patient (8%) is female. All patients had EMPD histology and were diagnosed as advanced EMPD. Primary tumor locations included vulva in 1 patient, scrotum in 8 patients, and penis in 3 patients. Metastatic sites included inguinal lymph nodes in all 12 patients, and noninguinal lymph nodes in 8 patients. Also, there are pulmonary metastases in 4 patients, liver metastases in 6 patients, and bone metastases in 7 patients. The median number of previous chemotherapy lines of treatment was 2 (range 1-6). Nine patients (75%) received prior surgery, and radiotherapy as a previous line of therapy for 6 patients (50%).

We found that camrelizumab in combination with rivoceranib was tolerated in this study with no immune-related adverse events reported. Unfortunately, this clinical trial did not show clinical activity for the combination in patients with advanced EMPD. All patients discontinued the combination therapy because of disease progression. One patient achieved stable disease for 9 months. The median PFS is 2.4 months, 95% CI, 2.1-4.4 months (Figure 1). Per study protocol, at least 2 objective responses were required to continue enrolling on the second stage of the study; therefore, the trial was closed at the completion of the first stage.

The main reason for the negative result may be the lack of PD-1/PDL1 expressions. Only one patient was identified as PD-L1 positive. In fact, there is still limited understanding of the relationship between EMPD and PD-1/PDL1 expression. A study evaluated PD-L1 expressions in 18 EMPD surgical pathology cases. PD-L1 was found in 3 cases (17%), including 2 of 4 invasive cases (50%) and 1 of 14 noninvasive cases (7%). Invasive cases with lymph node metastasis also showed PD-L1 expression. TAI cells had higher PD-L1 positivity (83%) compared to tumor cells (17%).^13^ Goto et al. investigated the relationship between EMPD and PD-L1/PD-1 expression in 39 Japanese EMPD patients using immunohistochemical staining. Results showed that Paget’s cells did not express PD-L1, but some tumor-infiltrating mononuclear cells (TIMCs) expressed PD-L1 and PD-1. However, no correlation was found between PD-L1/PD-1 expression in TIMCs and patient characteristics. The study concluded that further clinical research is needed to explore other immune escape pathways in EMPD.^14^ A study investigates the Warburg effect in EMPD, finding that elevated glycolytic enzymes like LDHA are associated with increased immunosuppressive cells and cytokines.^15^ These studies suggest that the tumor microenvironment of EMPD leads to immunotherapy resistance, suggesting that EMPD patients have a poor response to immunotherapy, which is consistent with our observation.

In conclusion, this is the first reported clinical trial evaluating carelizumab in combination with rivoceranib in advanced EMPD. This combination did not demonstrate clinical benefit in this cohort of patients with advanced EMPD. Further investigation requires evaluation of the mechanism of resistance and a possible new combination strategy.

## Data Availability

The data underlying this article will be shared on reasonable request to the corresponding author.
